# The Clinical Consistency and Utility of ICD-11 Diagnostic Guidelines for Gaming Disorder: A Field Study Among the Chinese Population

**DOI:** 10.3389/fpsyt.2021.781992

**Published:** 2021-12-22

**Authors:** Chenyi Ma, Zhe Wang, Chuanwei Li, Jing Lu, Jiang Long, Ruihua Li, Qianying Wu, Haifeng Jiang, Jiang Du, Runji Li, Peiyan Wang, Limin Ma, Hongwei Li, Shuqin Hui, Wenli Zhao, Na Zhong, Min Zhao

**Affiliations:** ^1^Shanghai Mental Health Center, Shanghai Jiao Tong University School of Medicine, Shanghai, China; ^2^The Affiliated Guangji Hospital of Soochow University, Jiangsu, China; ^3^UCLA College of Letters and Science, University of California, Los Angeles, Los Angeles, CA, United States; ^4^Lulong Vocational and Technical Education Center, Qinhuangdao, China; ^5^Shanghai Key Laboratory of Psychotic Disorders, Shanghai, China; ^6^Chinese Academy of Sciences (CAS), Center for Excellence in Brain Science and Intelligence Technology (CEBSIT), Chinese Academy of Sciences (CAS), Shanghai, China

**Keywords:** gaming disorder (GD), ICD-11 diagnostic guidelines, DSM-5 (the *Diagnostic and Statistical Manual of Mental Disorders*), the clinical consistency, clinical applicability and cultural adaption

## Abstract

**Purpose:** As a new category proposed in the *International Classification of Diseases* (11th Revision) (ICD-11), the reliability and clinical utility of ICD diagnostic guidelines for gaming disorder (GD) in the Chinese population have not been studied. The purpose of this field study is to clarify the reliability, clinical utility, and cultural applicability of ICD diagnostic guidelines for GD in China and its comparability with Internet GD (IGD) in the *Diagnostic and Statistical Manual of Mental Disorders* (Fifth Edition) (DSM-5).

**Methods:** Participants included 21 paired clinical raters consisting of seven psychiatrists and 200 gaming players aged from 15 to 18 years with different risk levels of Internet addiction based on the scores of Young's Internet Addiction Test. Each participant received a semi-structured face-to-face interview by paired clinical raters at the same time. Then clinical raters made the diagnosis and filled the clinical utility questionnaire independently according to the diagnostic guidelines for GD in both ICD-11 and DSM-5.

**Results:** The diagnostic consistency coefficient (kappa value) between the paired clinical raters was 0.545 (0.490–0.600, *p* < 0.001) and 0.622 (0.553–0.691, *p* < 0.001) for ICD-11 and DSM-5 diagnostic guidelines, respectively, for GD. The diagnostic consistency was 0.847 (0.814–0.880, *p* < 0.001) between GD in ICD-11 and IGD in DSM-5. Meanwhile, 86.7% of responses that agreed with the ICD-11 diagnostic guidelines for GD provided enough detailed implementation characteristics and showed good overall clinical applicability (86.0%), specificity (94.4%), usefulness (84.1%), and acceptable cultural adaptation (74.8%). GD in ICD-11 was slightly more accepted than IGD in DSM-5 (*p* < 0.001), while the clinical efficiency of ICD-11 was inferior to that of DSM-5 (*p* < 0.001).

**Conclusion:** This study indicates that the ICD-11 diagnostic guidelines for GD have acceptable clinical reliability and high consistency with IGD in DSM-5. Their clinical applicability and cultural adaption are comparable with those of DSM-5. Although the guidelines still need to be adjusted for better implementation in China, this is already a great step committed to reducing the serious consequences caused by excessive gaming behaviors through effective identification and normative diagnosis, especially for adolescents.

## Introduction

In 2018, the WHO released the draft of the 11th revision of the *International Classification of Diseases* (ICD-11) (1). It is worth noting that there is an inclusion of a new diagnostic category “gaming disorder (GD)” in the section “Disorders due to substance use or addictive behavior.” The decision was based on reviewing the available evidence in the scientific literature and on case series (2–4), as well as experiences from clinical practice provided by international experts in psychiatry, clinical psychology, internal medicine, family practice, epidemiology, neurobiology, and public health (5). This highly controversial diagnosis, thus, reminds us to pay more attention to problems caused by games and even the Internet (6).

GD is one of the “disorders due to addictive behaviors.” It has become a recognizable and clinically significant syndrome associated with distress or interference with personal functions that develop by repetitive rewarding behaviors other than the use of dependence-producing substances (1). National surveys have shown the prevalence rates of GD of 10~15% among young people in several Asian countries and of 1~10% in their counterparts in some Western countries (7).

In addition, recent studies confirmed that culture shapes gaming-related distress and partially influences the psychiatric presentation of gaming-related distress symptoms (7, 8). Due to the differences between Eastern and Western cultures, there will be differences in the types and content of games that players prefer, which will surely have a greater impact on gaming behaviors. Unrestricted gaming may firmly attract users' attention and become addictive for vulnerable individuals (9, 10). It is confirmed that children and adolescents have the greatest risk of GD and that excessive gaming behavior is considered to be a serious social problem (11). From this, corresponding diagnostic guidelines are essential to facilitate accurate diagnosis and to prevent discrimination against gaming behavior, inappropriate diagnosis of GD, or even unnecessary therapeutic interventions (12, 13). Including this new diagnostic category in ICD-11 is indeed a timely response to social needs. The establishment of GD is not a denial of gaming but an emphasis on health problems caused by problematic gaming behaviors.

With increasing attention and in-depth research, GD shares many similarities in clinical and neuroimaging characteristics with substance use disorder and gambling disorder. Recent studies show that young people with Internet GD (IGD; diagnosed by DSM-5) exhibited significantly blunted neural responses within distributed subcortical and cortical regions including the striatum, insula, lateral prefrontal cortex, and anterior cingulate in response to negative affective cues, as well as in the process of emotional regulation (14–16). Recovered IGD subjects showed lower dorsolateral prefrontal cortex (DLPFC) activation than persistent IGD subjects to gaming cues at both pre-gaming and post-gaming times (17). Relatively decreased DLPFC activity and increased insula activity in response to gaming cues following recent gaming may underlie the persistence of gaming (17).

In response to this increasingly serious problem, the proper diagnosis of GD is vitally important for targeted interventions and treatments. In other words, classifying GD as a disorder does not deny the existence of games themselves but rather is a step toward early intervention in excessive game use (11). For this reason, detailed diagnostic criteria are proposed in the draft of ICD-11. Three essential features are required, including (1) a pattern of persistent or recurrent gaming behavior, (2) an extended period (e.g., 12 months), and (3) marked distress or significant impairment in important domains of functioning (1). Moreover, the draft of ICD-11 also includes many other additional features and boundaries with other disorders and conditions to better understand the diagnostic criteria and then make an appropriate diagnosis.

As a new diagnostic category, GD is now poorly understood, and no data exist on the reliability and validity of ICD-11 diagnostic guidelines based on the Chinese population. So the guidelines need to be verified urgently before being applied in Chinese clinical practice.

Before the inclusion of GD in ICD-11, problematic gaming behaviors were generally identified based on the diagnostic criteria of IGD in DSM-5 updated in 2013. Therefore, the diagnostic consistency of ICD-11 and DSM-5, which can affect the accuracy of clinical practice, has not been confirmed yet. Most studies that focused on GD relied only on questionnaire material instead of clinical interviews. On the other hand, there is still a lack of researches to collect clinicians' preference for GD in ICD-11 or IGD in DSM-5.

In order to test the reliability, clinical utility, and cultural applicability of ICD-11 diagnostic guidelines for GD in China, field testing was designed on the diagnostic consistency and clinical utility among well-trained clinical raters toward excessive gaming players. We hypothesized first that the clinical consistency of ICD-11 diagnostic guidelines for GD would be as high as that of DSM-5. We expected GD in ICD-11 and IGD in DSM-5 to be highly consistent in identifying problematic gaming behaviors. Furthermore, this study would demonstrate the advantages and disadvantages of ICD-11 new diagnostic guidelines for GD in the diagnosis compared with IGD in DSM-5.

## Methods

A field study of GD was conducted from August 2019 to January 2020 at two sites in China, including Shanghai and Hebei. The study obtained a concurrent, joint-rater agreement on the diagnosis and clinical utility of the ICD-11 diagnostic guidelines for GD. Paired clinical raters conducted clinical interviews with gaming players to determine whether the players met the diagnostic criteria of GD in ICD-11 or IGD in DSM-5. And then the raters evaluated the clinical utility of the diagnostic guidelines through online diagnosis and evaluation questionnaires.

**Figure 1:**
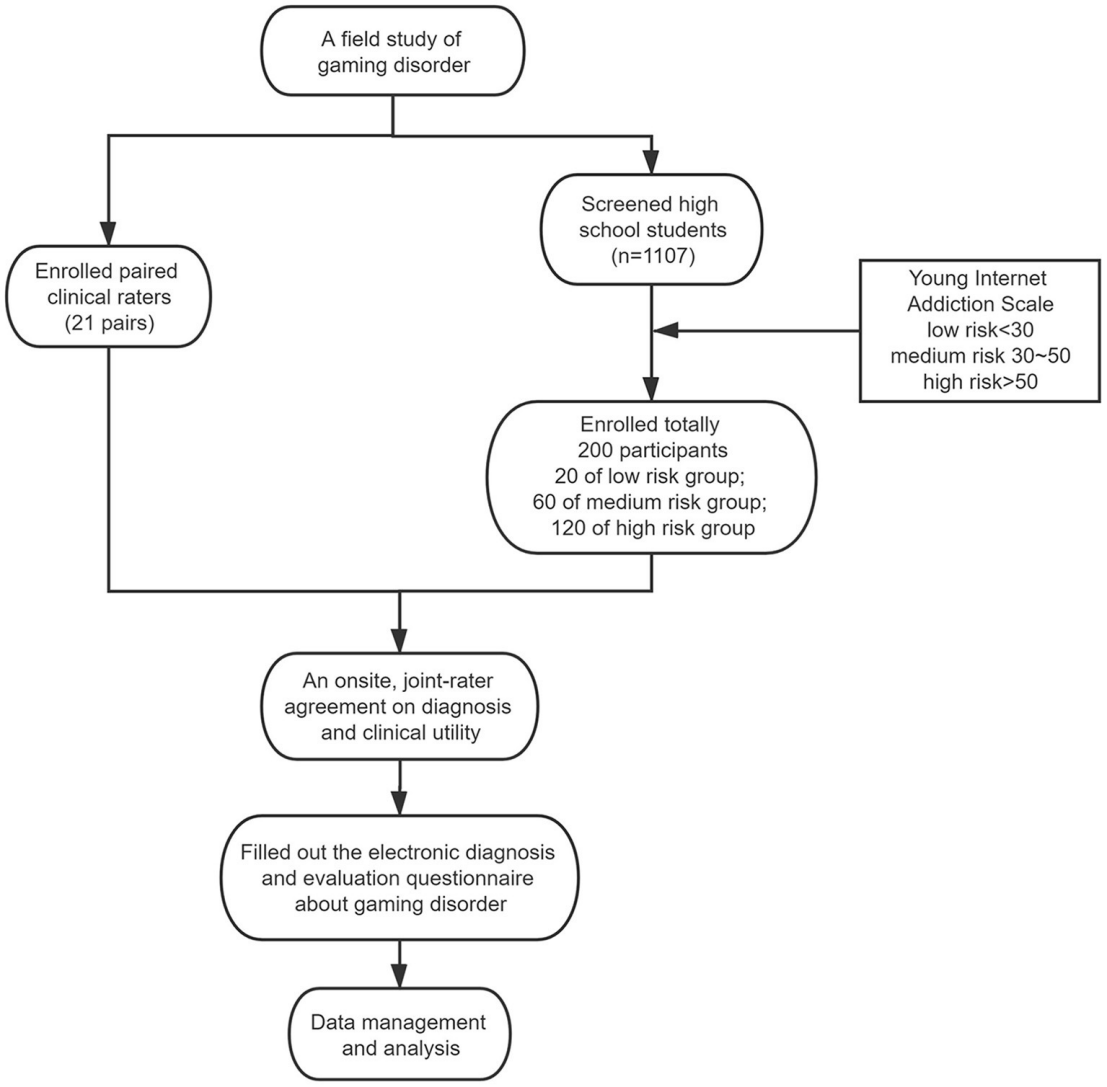
Study flowchart.

The study flowchart is shown in Figure 1.

### Procedure

#### An Onsite, Joint-Rater Agreement on Diagnosis and Clinical Utility of Gaming Disorder

Clinical raters were numbered serially when recruiting and were paired randomly according to their unique numbers. Similarly, gaming players also had their numbers and were randomly assigned to a pair of clinical raters. Then, 21 possible pairings of clinicians assigned a certain number of gaming players to interview jointly and judged them on whether they met the criteria of GD in ICD-11 or IGD in DSM-5. One clinician acted as a major interviewer (clinical rater I) and the other as an observer (clinical rater II). Clinician pairings varied as much as possible, and participating clinicians alternated as primary interviewer and observer. This would prevent clinician pairings from converging in diagnosis and overestimating the consistency of the diagnostic guidelines. At the end of the interview, the observer was allowed to ask additional questions (18).

Each clinician drew a diagnosis independently based on interviews and, sometimes, additional supplementary information (for example, comments from their teachers and roommates). That is to say, each gaming player involved in this study had two raters for clinical assessment, and each clinical rater would draw a diagnosis based on ICD-11 and DSM-5. After that, clinical raters filled out a basic information form and an interview list for details and scores on the clinical utility of ICD-11 GD diagnostic guidelines.

### Participants

#### Excessive Gaming Players

There were 1,107 students aged 15 to 18 recruited from a vocational and technical education center in Hebei province, and they were invited to complete Young's Internet Addiction Test (IAT). Based on the IAT scores, students were divided into three groups: high risk of Internet addiction (>50), medium risk (30–50), and low-medium risk (<30). Finally, a total of 200 gaming players (120 students of a high-risk group, 60 of a medium-risk group, and 20 of a low-medium group) were willing to join the face-to-face clinical interview by stratified random sampling based on their IAT scores. The inclusion criteria were as follows: (1) the players were voluntarily willing to participate in the study, and both the teens and their guardians gave signed informed consent; and (2) they have normal or corrected to normal sight, and normal hearing.

#### Clinical Raters

A total of 21 pairs of clinicians paired randomly by seven psychiatrists were recruited from Shanghai and Suzhou. The recruitment was mainly carried out in our hospital and other top mental health centers in surrounding areas. We welcomed as many excellent clinicians as possible to join our research, especially those who were specialists in addiction and interested in ICD-11 diagnostic guidelines for GD. They took part in the intensive training organized on the ICD-11 diagnostic guidelines for GD and DSM-5 diagnostic criteria for IGD for at least five class hours. After that, they all passed a test consisting of two standardized cases to confirm that they had already built a correct understanding of GD. The inclusion criteria of clinical raters were as follows: (1) at least 3-year working experience in clinical settings, (2) willing to receive training on the diagnostic guidelines for GD in ICD-11 and DSM-5, (3) specializing in psychiatry and having the opportunity to encounter individuals with problematic gaming behaviors at work, and (4) willing to participate in the study voluntarily.

### Measurements

#### Young's Internet Addiction Test

Young's IAT was used as a screening tool that could help quickly screen individuals with potentially problematic gaming behaviors for clinical interviews. This self-report tool targets the present addictive Internet behaviors and its Chinese version with good validity and reliability (19). It contains 20 items and can be completed in about 7 min. Examinees need to respond to each item with a number between 1 and 5. The total scores range from 20 to 100, indicating the extent to which they endorse those particular behaviors. In this study, three groups of participants divided by scores of IAT were high-risk group (>50), medium-risk group (30~50), and low-risk group (<30).

#### Online Diagnosis and Evaluation Questionnaires on Gaming Disorder

The paired clinical raters need to separately complete an online questionnaire. The questionnaire consists of the following two parts: (1) diagnosis part. The clinical raters must judge whether the game player met the individual essential features and additional features based on ICD-11 diagnostic guidelines for GD so that they could draw the final diagnosis of GD, hazardous gaming, or neither. And they needed to further diagnose the presence or comorbidity of other mental disorders such as substance use disorder, bipolar disorder, and attention-deficit hyperactivity disorder. After that, they also needed to judge each of the nine diagnostic criteria of DSM-5 and then reach a diagnosis of IGD or not. (2) Evaluation of the clinical applicability of ICD-11 diagnostic guidelines for GD. It was assessed on five aspects of clinical applicability, implementation characteristics, specificity, usefulness, and cultural adaptation. According to the actual situation of every subject interviewed, clinical raters could choose one of the five options (not at all, somewhat, moderate, quite, or extremely) to evaluate the clinical applicability of GD in ICD-11. (3) Comparison of clinical utility ratings for ICD-11 GD and DSM-5 IGD. There were a total of nine items. And each item had a full score of 10 points. Same as above, both GD in ICD-11 and IGD in DSM-5 were evaluated in terms of core clinical utility questions, implementation characteristics of the guidelines, the utility of specific sections of the guidelines, and the utility of the guidelines for specific purposes.

### Data Management and Analysis

All analyses were conducted using SPSS. To estimate diagnostic reliability, kappa coefficients for diagnoses were calculated followed by bootstrapping 95% CIs for kappa. Landis and Koch adjectives were used to describe ranges of reliability values for kappa: slight (from 0 to 0.20), fair (from 0.21 to 0.40), moderate (from 0.41 to 0.60), substantial (from 0.61 to 0.80), and almost perfect (from 0.81 to 1.0) (20). A descriptive statistical method was used to calculate the frequency of response and sample features. The paired-samples *t*-test was used to compare the clinical utility ratings for GD in ICD-11 and IGD in DSM-5 (α = 0.05).

All the participants were fully informed of the purpose of the study and the process, signed the informed consent, and promised not to disclose the privacy of any participant. If the participants were under 18 years old, their guardians were fully informed and then signed the informed consent for them. The study was conducted in accordance with the Declaration of Helsinki and approved by the Ethics Committee of Shanghai Mental Health Centre (ethics approval number: 2019-73).

### Role of Funding Source

The funder had no role in study design, data collection, data analysis, data interpretation, or writing of the report. The corresponding authors (MZ and NZ) had full access to all the data in the study and had final responsibility for the decision to submit for publication.

## Results

This study recruited a total of seven psychiatrists, aged 31.7 ± 6.4 years, with a bachelor's degree and above. They were all from top mental health centers in Shanghai and Suzhou. They had the qualifications of the clinical psychiatrist in China, with an average working experience of 7.3 ± 4.8 years.

According to the scores of Young's IAT, 395 students were divided into the low-risk group (<30), 576 in the medium-risk group (30–50), and 127 in the high-risk group (>50), and nine invalid questionnaires were excluded. Twenty (5.1% of the low-risk group), 60 (10.4% of the medium-risk group), and 120 (94.5% of the high-risk group) participants were included. Finally, 200 students participating in the study were 16.5 ± 1.0 years old. And most of them were male (85.6%).

### The Diagnostic Consistency of *International Classification of Diseases* (11th Revision) Diagnostic Guidelines for Gaming Disorder

Table 1 shows the results diagnosed by ICD-11 diagnostic guidelines for GD. The diagnosis of 158 (78.0%) subjects was consistent between the paired clinical raters, of which 29 (14.5%) were GD, 8 (4.0%) were hazardous gaming, and 119 (59.5%) were neither GD nor hazardous gaming (Table 1). The diagnostic consistency coefficient (kappa value) between paired clinical raters was 0.545 (0.490–0.600, *p* < 0.001), which was considered moderate reliability according to Landis and Koch's adjectives.

**Table 1:** Diagnosis based on ICD-11 diagnostic guidelines for gaming disorder.

		**Clinical rater I**			
**Number of subjects**		**Gaming disorder**	**Hazardous gaming**	**Neither**	**Total**
Clinical rater II	Gaming disorder	29	10	18	57
	Hazardous gaming	0	8	7	15
	Neither	0	9	119	128
	Total	29	27	144	200

*ICD-11, International Classification of Diseases (11th Revision)*.

### The Diagnostic Consistency of *Diagnostic and Statistical Manual of Mental Disorders* (Fifth Edition) Diagnostic Criteria of Internet Gaming Disorder

The diagnostic consistency coefficient of DSM-5 diagnostic criteria of IGD was 0.622 (0.553–0.691, *p* < 0.001), suggesting that paired clinical raters had good diagnostic reliability based on the diagnostic criteria of IGD in DSM-5 (Table 2).

**Table 2:** Diagnosis based on DSM-5 diagnostic criteria of Internet gaming disorder.

	**Clinical rater I**			
**Number of subjects**		**Internet gaming disorder**	**No**	**Total**
Clinical rater II	Internet gaming disorder	27	20	47
	No	4	149	153
	Total	31	169	200

*ICD-11, International Classification of Diseases (11th Revision)*.

### The Diagnostic Consistency of the Diagnosis for Gaming Disorder in *International Classification of Diseases* (11th Revision) and Internet Gaming Disorder in *Diagnostic and Statistical Manual of Mental Disorders* (Fifth Edition)

The diagnostic results were divided into two categories, GD and not. All subjects diagnosed as “hazardous gaming” or “neither” according to ICD-11 were considered to be inconsistent with the diagnosis of GD (Table 3). The consistency test suggested that the kappa value was 0.847 (0.814–0.880, *p* < 0.001). For the same subject, the diagnostic consistency of GD in ICD-11 and IGD in DSM-5 made by the same clinical rater was almost perfect.

**Table 3:** Diagnosis based on ICD-11 GD and DSM-5 IGD.

		**Diagnosis based on DSM-5**		
		**Internet gaming disorder**	**No**	**Total**
Diagnosis based on ICD-11	Gaming disorder	72	14	86
	No^a^	6	308	314
	Total	78	322	400

*ICD-11, International Classification of Diseases (11th Revision); DSM-5, Diagnostic and Statistical Manual of Mental Disorders (Fifth Edition)*.

a *All subjects who were diagnosed as hazardous gaming or neither according to ICD-11 were considered to be inconsistent with the diagnosis of gaming disorder*.

### The Clinical Utility of *International Classification of Diseases* (11th Revision) Diagnostic Guidelines for Gaming Disorder From the Onsite Interview

There are 400 sets of clinical utility ratings because there were two raters for each gaming player (*N* = 200).

In general, the present data indicated that most participants agreed with the excellent clinical applicability (86.0%), detailed enough implementation characteristics (86.7%), high specificity (94.4%), great usefulness (84.1%), and good cultural adaptation (74.8%) of ICD-11 diagnostic guidelines for GD by rating “quite” or “extremely.”

**Figure 2:**
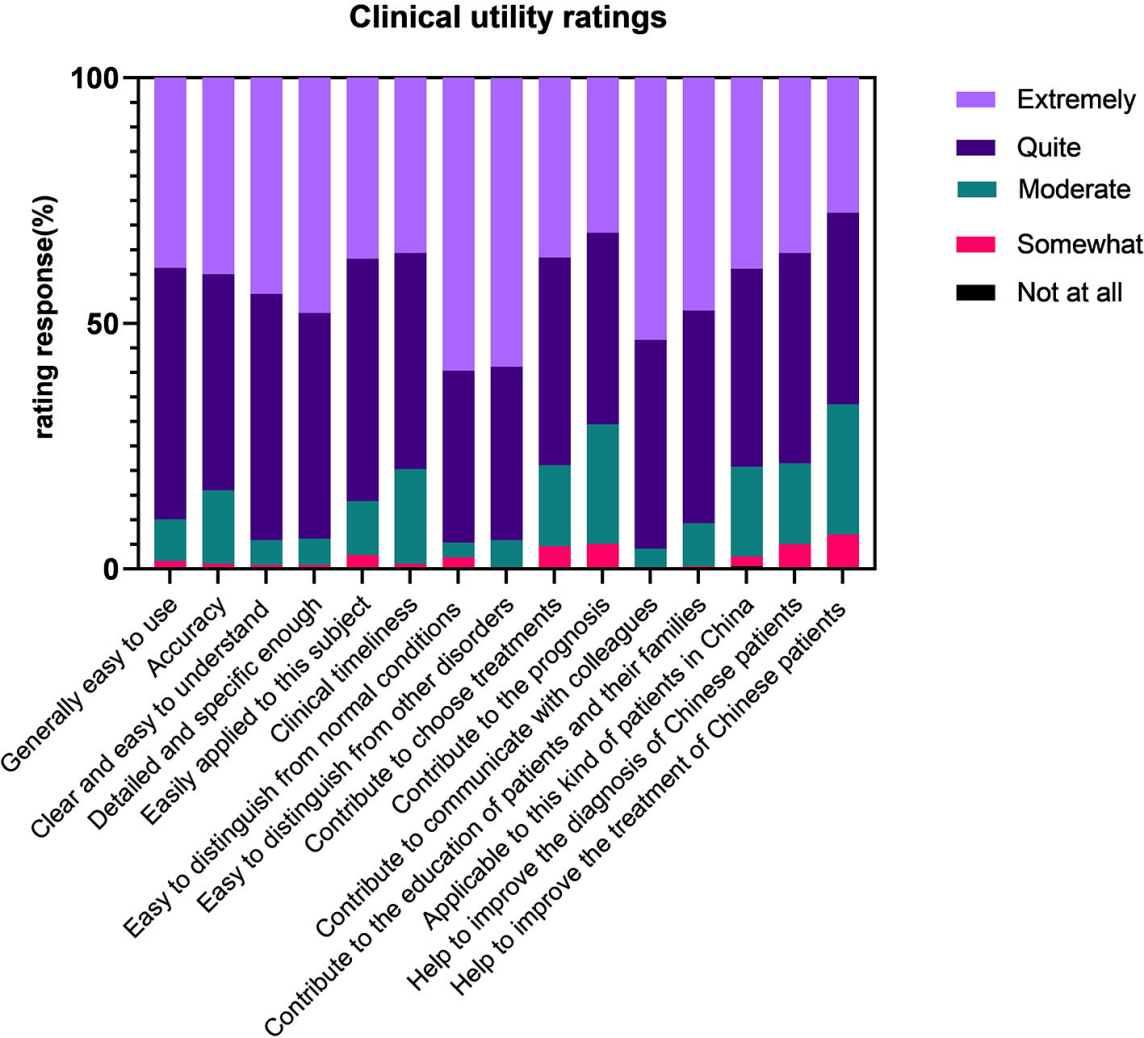
Clinical utility ratings for the diagnosis of the *International Classification of Diseases* (11th Revision) (ICD-11) gaming disorder.

In detail (Figure 2), the vast majority of participants agreed with the guidelines' excellent clinical applicability by providing ratings of “quite” or “extremely,” which meant easy to use (90.0%), accuracy (84.0%), and clear and understandable (84.0%).

For implementation characteristics, most responses showed that guidelines were detailed and specific enough (94.0%), easily applied to individuals (86.3%), and cost the same or even less time than usual (79.8%).

Also, the diagnostic guidelines had enough information to identify GD from normal conditions (94.8%) and other mental disorders (94.0%).

Considering the usefulness, a large number of participants indicated that the guidelines could contribute to choosing treatments (79.0%), to the prognosis (70.8%), to communicating with colleagues (96.0%), and to the education of patients and their families (90.8%).

In the last part regarding the cultural adaption in China, still many responses supported that the guidelines are applicable to this kind of patients in China (79.3%) and help to improve the diagnosis (78.5%) and the treatment of Chinese patients (66.5%).

Overall, ICD-11 diagnostic guidelines for GD were slightly more acceptable than those for IGD in DSM-5 (*p* < 0.001), except for the aspect of “clear and easy to understand” (*p* = 0.057). However, the clinical efficiency of ICD-11 was inferior to that of DSM-5 (*p* < 0.001) (Table 4).

**Table 4:** A comparison of clinical utility ratings for GD in ICD-11 and IGD in DSM-5.

**Mean rating (SD)**	**ICD-11**	**DSM-5**	**t**	***p*** **-Value**
Easy to use	8.16 (1.426)	7.90 (1.536)	5.287	<0.001**
Accuracy of diagnosis	8.47 (1.214)	7.79 (1.643)	12.787	<0.001**
Clear and easy to understand	8.16 (1.526)	8.04 (1.437)	1.906	0.057
Sufficient detail and specificity	8.33 (1.353)	7.62 (1.571)	13.463	<0.001**
The clinical efficiency	7.81 (1.532)	8.05 (1.579)	−3.738	<0.001**
Contribute to choose treatments	7.83 (1.717)	7.65 (1.781)	4.690	<0.001**
Contribute to the prognosis	7.80 (1.735)	7.57 (1.746)	5.946	<0.001**
Contribute to communicate with colleagues	7.86 (1.703)	7.71 (1.749)	4.355	<0.001**
Contribute to the education of patients and their families	7.79 (1.707)	7.57 (1.759)	6.175	<0.001**

*GD, gaming disorder; ICD-11, International Classification of Diseases (11th Revision); IGD, Internet gaming disorder; DSM-5, Diagnostic and Statistical Manual of Mental Disorders (Fifth Edition)*.

** *p < 0.001*.

## Discussion

This study mainly focused on the reliability and clinical utility of ICD-11 diagnostic guidelines for GD compared with IGD in DSM-5 based on clinical data in China. The results show moderate diagnostic consistency of ICD-11 diagnostic guidelines for GD. And the results were slightly inferior to those of DSM-5. As we expected, the diagnosis based on two different diagnostic systems by the same clinical rater was highly consistent. Meanwhile, this study indicated high clinical utility, good cultural adaption in China, and excellent comparability with DSM-5. The result implied that the new diagnostic guidelines for GD were highly reliable, helpful, and appropriate for the Chinese population and healthcare system.

First of all, the study indicated moderate diagnostic consistency of the ICD-11 guidelines for GD between clinical raters. Moderate diagnostic consistency was probably a result of the unified training of these psychiatrists on diagnostic guidelines for GD at the beginning of this study. And it suggested that the use of more uniform procedures by clinicians based on brief training might yield adequate reliability for this new GD (20). This concurrent reliability of ICD-11 diagnoses of GD was not substantial as other ICD-11 diagnoses of mental disorders with high burdens such as schizophrenia, mood disorder, and stress-related disorder (21). As a new category, most clinical practitioners were unfamiliar with the clinical features of GD and have limited experience with these excessive game players. So more proper training on GD should be provided to clinical practitioners to help related workers develop consistency and familiarity with GD.

Besides, the clinical raters were quite consistent with the diagnoses of hazardous gaming in the chapter of problems associated with health behaviors in the ICD-11. Hazardous gaming is also a critical change in ICD-11. It refers to a pattern of gaming that increases the risk of harmful physical or mental health to individuals, which raises a potential need for prevention interventions away from the development of GD (1). It will help prevent occurrences like the reported game-playing-associated death of a hospitalized patient and facilitate the development of prevention and treatment efforts (22). There seems to be a progressive relationship between hazardous gaming and GD, and the boundary is blurry. This undoubtedly provides the clinician with greater flexibility. So they can diagnose according to the actual situation and will also benefit greatly the groups with a high risk of GD regarding prevention and intervention. But the clinical raters might find it hard to develop a comprehensive and profound understanding of hazardous gaming. They lacked confidence in drawing such a new diagnosis. For this reason, systematic training could effectively improve the accuracy of clinical practice.

However, still, some objective factors may cause negative effects on the diagnostic consistency. This study was conducted in a vocational and technical education center instead of clinical settings. So the number of adolescents with serious problematic gaming behaviors was relatively small. Since they were from the same boarding school in the same area, their life and learning background had some common characteristics that might influence their gaming behaviors. For instance, most of them chose mobile phones as their medium and mainly played online games. And their games were restricted on weekdays because their school had strict regulations on mobile phones.

As for DSM-5, the consistency of DSM-5 diagnostic criteria for IGD was slightly superior to that of ICD-11. IGD was proposed earlier than GD in ICD-11. In recent years, both clinical practice and academic researches on problematic gaming behaviors have referred to DSM-5 diagnostic criteria, leading to higher acceptance and mastery as well as higher reliability in diagnosis. Meanwhile, IGD in DSM-5 provides 9 clear diagnostic criteria, and the diagnosis can be made as long as 5 of them are met. These criteria are more specific and undoubtedly have higher operability in clinical practice.

The concurrent reliability of two different diagnostic systems, ICD-11 and DSM-5, was high enough. This was mainly due to high content overlap between 3 essential features of ICD-11 for GD and 9 diagnostic items of DSM-5 for GD. It was exactly in line with our expectations (23). That is to say, two diagnostic systems had a unified understanding and description of such kind of disorder so that they could accurately identify problematic gaming behaviors for future clinical practice and academic researches. These results are consistent with the previous research. Ko et al. also supported the utility of ICD-11 and DSM-5 in identifying individuals who need treatment for problematic gaming behaviors (24).

Besides the diagnosis, the clinical utility of ICD-11 diagnostic guidelines for GD is another focus of this study. As we expected, they showed high clinical value during diagnosis. In most cases, clinical psychiatrists agreed that diagnostic guidelines were highly accurate, generally understandable, and easy to apply. In addition to essential and additional features, the guidelines have an excellent part that prompts the identification of GD from normality and other disorders to improve accuracy and specificity. Also, they were perceived as roughly suitable in China in terms of cultural adaptability whether in diagnosis or communication with colleagues and patients.

In consideration of better implementation in China, ICD-11 diagnostic guidelines for GD still need to be adjusted according to the Chinese culture and healthcare system. For professionals in high-incidence settings of GD, such as schoolteachers, pediatric clinicians, and psychological workers, more training and education about the features and prevention strategies are needed. Also, most clinical raters mentioned that the proposed course of GD was 12 months, but the required duration may be shortened if all diagnostic requirements are met and the patients' symptoms are severe. These provide clinicians certain flexibility to different cultures or cases, but they might also bring some confusion leading to different diagnostic results among clinical workers. So developing some supplementary instruction to diagnostic guidelines suitable for the Chinese culture would make the diagnosis more rigorous and exact.

Still, this study has some limitations. First, the current study accessed only a relatively small proportion, and the number of clinical raters involved was small. Each rater pair interviewed several to more than 10 gaming players, subjected to their working hours, research location, and conditions. Although we chose individuals who were more likely to be troubled by excessive gaming behaviors, samples were still relatively single and all from the same school. This might bring bias in the utility of the diagnostic guidelines. On the other hand, further training on GD should be conducted for more clinicians and other related staff to get a comprehensive understanding of ICD-11 diagnostic guidelines for GD. Second, the concurrent method of testing reliability, drawing on the field study of ICD-11 (25), usually generates higher kappa values because the two diagnosticians obtain nearly the same information during the interview (26). Moreover, this study was conducted in the gaming players' school, and all questionnaires and interviews were self-reported. So students might have concerns and hold back some information. Thus, further studies need to be conducted to collect more data from different populations and hear more relevant professionals' opinions on ICD-11 diagnostic guidelines for GD.

## Conclusion

On the whole, the present data indicated moderate reliability of ICD-11 diagnostic guidelines for GD and their acceptable clinical and public health values as well as good cultural adaption as compared with DSM-5 guidelines. Meanwhile, GD in ICD-11 and IGD in DSM-5 had high concurrent reliability as we expected. Correct diagnosis could undoubtedly reduce the serious consequences caused by excessive gaming behavior, especially for adolescents. In the future, adequate training to promote ICD-11 diagnostic guidelines is essential for excellent clinical practice. More research is recommended to explore pathogenesis, risk factors, interventions, and even prognosis for a more comprehensive understanding of GD or even other disorders caused by Internet overuse.

## Data Availability Statement

The raw data supporting the conclusions of this article will be made available by the authors, without undue reservation.

## Ethics Statement

The studies involving human participants were reviewed and approved by the Ethics Committee of Shanghai Mental Health Centre. Written informed consent to participate in this study was provided by the participants' legal guardian/next of kin.

## Author Contributions

MZ, NZ, and CM conceived the idea and designed the study. WZ and CL contributed for data management. CM, ZW, CL, and JLu analyzed and interpreted the data. MZ and NZ contributed for quality control. CM wrote the first version of the manuscript. All authors helped recruiting the participants, collecting data, conceptualization, study design, data-collection, data management, data analysis, interpretation, writing, critically reviewed, and approved the final version of the manuscript.

## Funding

This work was supported by National Nature Science Foundation (81771436, 82130041, and 82171485), Shanghai Municipal Science and Technology Major Project (2018SHZDZX05), Shanghai Shenkang Hospital Development Center (SHDC2020CR3045B), Shanghai Clinical Research Center for Mental Health (19MC1911100), Shanghai Engineering Research Center of Intelligent Addiction Treatment and Rehabilitation (19DZ2255200), Shanghai Key Laboratory of Psychotic Disorders (13DZ2260500), Shanghai Mental Health Center Clinical Research Center (CRC 2018YB02), and Shanghai Municipal Health Commission(20184Y0134).

## Conflict of Interest

The authors declare that the research was conducted in the absence of any commercial or financial relationships that could be construed as a potential conflict of interest.

## Publisher's Note

All claims expressed in this article are solely those of the authors and do not necessarily represent those of their affiliated organizations, or those of the publisher, the editors and the reviewers. Any product that may be evaluated in this article, or claim that may be made by its manufacturer, is not guaranteed or endorsed by the publisher.
